# The Plasticity of Genome Architecture

**DOI:** 10.3390/genes11121413

**Published:** 2020-11-27

**Authors:** Marta Farré, Aurora Ruiz-Herrera

**Affiliations:** 1 School of Biosciences, University of Kent, Canterbury CT2 7NJ, UK; m.farre-belmonte@kent.ac.uk; 2 Genome Integrity and Instability Group, Institut de Biotecnologia i Biomedicina, Universitat Autònoma de Barcelona, 08193 Cerdanyola del Vallès, Spain; 3 Departament de Biologia Cel.lular, Fisiologia i Immunologia, Universitat Autònoma de Barcelona, Campus UAB, 08193 Cerdanyola del Vallès, Spain

Understanding the origin of species and their adaptability to new environments is one of the main questions in biology. This is fuelled by the ongoing debate on species concepts and facilitated by the availability of an unprecedented large number of genomic resources. Genomes are organized into chromosomes, where significant variation in number and morphology is observed among vertebrates [1] due to large-scale structural variants, such as inversions, translocations, fusions, and fissions. This genomic reshuffling provides, in the long term, new chromosomal forms on which natural selection can act upon. Additionally, reorganizations can also trigger the development of inherited diseases by altering gene expression and regulation of the affected genomic regions. In this context, the characterization of genome plasticity among taxa will provide fertile grounds for exploring the dynamics of genome composition, the evolutionary relationships between species, and in the long run, speciation.

This Special Issue includes mainly articles, reviews, and an opinion piece that explore one or more of the themes mentioned above. From the chromosomal characterization of species to the study of sex determination, including the dissection of satellite DNA as drivers of genome evolution, sex chromosome evolution, and the dynamics of chromosomal reorganizations in germ cells. With this as framework, Deakin and collaborators [2] propose the adoption of the term “chromosomics”, stressing the need to integrate both genomic and cytogenetic data to provide a comprehensive view of the role genome architecture in genome plasticity. In their work, the authors review how cytogenetics is an integral part of large-scale genome projects, highlighting the big questions in genome biology requiring a chromosomics approach. In fact, high-quality reference genome assemblies are fundamental for the application of genomics to biology, disease, and biodiversity conservation [3]. Altogether, the characterization of karyotypes is of paramount importance in this field.

New genome sequencing efforts are now in place to obtain telomere-to-telomere sequences for any given chromosome [4], yet only a handful of human chromosomes are currently at this level. Sequencing and assembling the repetitive fraction of the genomes, particularly large tandem repeats and satellite DNA (satDNA), is still an ongoing issue for most vertebrate and invertebrate species. In their paper, Lousada et al. [5] review current approaches combining state-of-the-art sequencing with molecular cytogenetics and optical mapping to investigate satDNA. The authors discuss the dynamic behaviour of satDNA within and between species and the role it might play in genome plasticity and chromosome rearrangements. A clear example of how satDNAs are widely variable among closely related species is shown by Sember and colleagues [6]. In this publication, focusing on two species of bighead carps (genus *Hypophthalmichthys*), the authors show that their taxonomic diversity is not associated with chromosome rearrangements; instead, their karyotypes can only be distinguished by the distribution of heterochromatin, a type of satDNA, among other repetitive elements. Conversely, in the neotropical *Nannostomus* pencilfishes, Sember et al. [7] show that a clear karyotype differentiation due to centric fusions exists, with little variation in repetitive DNA, therefore pointing to two different mechanisms of genome evolution in these two groups of fishes.

Several animal clades show high karyotype stability, such as birds, with most of the species presenting a diploid number of 2n = 80 [1]. Studying Gruiformes, a clade where most species conform to the typical avian karyotype, De Oliveira et al. [8] use comparative chromosome painting with chicken macro-chromosome paints to investigate karyotype evolution within the group. Then they used the few inter-chromosomal rearrangements detected to reconstruct a highly resolved phylogenetic relationship among these species, suggesting that karyotype diversification is a useful marker to separate gruiform species. In contrast, focusing on Columbidae birds, an avian clade with a wider range of diploid numbers, Kretschemer and colleagues [9] make use of chromosome paintings and bacterial artificial chromosome (BAC) clones to not only identify inter-chromosomal rearrangements, but also new intra-chromosomal rearrangements in large chromosomes. Mirroring previous studies (i.e., [10]), the authors show that micro-chromosomes are maintained as syntenic blocks in columbid birds.

Differences in karyotype number and morphology have not only been studied between species, but also within species. In this context, Garcia and colleagues [11] focus their efforts on the investigation of karyotype diversity in several genetically differentiated populations of the lesser white-toothed shrew (*Crocidura suaveolens*). They demonstrate that chromosomal rearrangements in the form of chromosome fusions are associated with increased genetic isolation among these shrew populations. Yet, another source of genome variation is exemplified by Robertsonian (Rb) fusions, which involves the centric fusion of two acrocentric chromosomes to form a single metacentric [12]. Rb fusions represent one of the principal sources of karyotype variation, as they are present in different taxa including mammals, reptiles, and insects. These include small mammals such as the house mouse (*Mus musculus domesticus*), whose natural populations present Rb fusions at a high rate [13,14,15]. In their paper, Tapisso et al. [16] provide an overview of the spatial and temporal dynamics of contact zones between chromosomal races of the house mice on the island of Madeira, which involves an extraordinary chromosomal variability and includes six metacentric races with diploid numbers ranging from 22 to 38. They provide insights into the dynamic processes that govern chromosomal variation at these contacts between chromosomal races. The authors propose that different interacting mechanisms such as landscape resistance, behaviour, chromosomal incompatibilities, and meiotic drive may help to explain the observed patterns.

Likewise, Rb fusions can have an impact on fertility, mainly attributed to the presence of defective chromosome synapsis and reduction in recombination [17,18]. This way, Rb fusions have often been invoked in the development of chromosomal incompatibilities between divergent lineages, thus contributing to chromosomal speciation [19,20]. In this context, Matveevsky and collaborators [21] propose the model of “contact first in meiosis” to explain the emergence of Rb fusions in meiosis. For that, the authors make use of the species *Ellobius alaicus*, mole voles that are characterized by (i) a high variability of diploid numbers due to Rb fusions and (ii) the presence of atypical sex chromosome systems.

In fact, sex chromosomes represent one of the most dynamic parts of the genome. There is an extremely high diversity among taxa in terms of sex chromosome number and morphology. Such diversity is exemplified by the five contributions on the evolution of sex chromosomes included in this Special Issue. Starting with insects, Sharma and collaborators [22] examine similarities and differences in heterochromatin patterns within X mitotic chromosomes among the major malaria vectors *Anopheles gambiae*, *An. coluzzii*, *An. arabiensis*, minor vector *An. merus*, and zoophilic non-vector *An. quadriannulatus*. Combining fluorescence in situ hybridization (FISH) with ribosomal DNA (rDNA), a highly repetitive fraction of DNA, and heterochromatic bacterial artificial chromosome (BAC) clones, the authors identify differences in the size and structure of the X chromosome heterochromatin, suggesting the possible role of repetitive DNA in the speciation of mosquitoes.

Sex determination in Sauropsids (reptiles and birds), on the other hand, is rather complex as Bista and Valenzuela [23] present in their review. The authors provide insights into the evolution of the reptilian karyotype and the genomic architecture of sex determination. They present an overview on the karyotypic changes that have accompanied the evolution of chromosomal systems of genotypic sex determination (GSD) in chelonians from systems under the control of environmental temperature (TSD). Overall, authors suggest that turtles have followed some tenets of classic theoretical models of sex chromosome evolution, while countering others. Birds, on the other hand, show distinctive patterns of sex chromosome degeneration, as Yazdi and collaborators [24] highlight in their opinion piece. Different forces have reshaped gene content of sex chromosomes, including Muller’s ratchet, or genetic hitchhiking/background selection [25], although other mechanisms have also been proposed [26]. In their work, Yazdi and colleagues [24] explore why some sex chromosomes degenerate more slowly than others among birds. To do so, authors analyse selective and neutral processes involved in recombination suppression and chromosome degeneration during sex chromosome evolution, using the largely recombining ancient sex chromosomes of ratites as a case study.

As for sex chromosome evolution in mammals, Romanenko and collaborators [27] present the intriguing case of the mandarin vole (*Lasiopodomys mandarinus*), characterized by a complex sex chromosome system (neo-Xs). In their study, the authors provide a comprehensive view of this species combining conventional and molecular cytogenetic methods, single chromosome DNA sequencing, and breeding experiments, revealing the chromosome segregation pattern as well as the reproductive performance of different karyomorphs. Finally, Proskuryakova et al. [28] illustrate how, by comparative chromosome mapping, it is possible to identify variations in the X chromosome structure of four bovid species: nilgai bull (*Boselaphus tragocamelus*), saola (*Pseudoryx nghetinhensis*), gaur (*Bos gaurus*), and Kirk’s Dikdik (*Madoqua kirkii*).

In conclusion, the papers in this Special Issue show the importance of combining the use of different methodological approaches to provide an overview of the dynamic and plasticity of genome architecture not only in vertebrates (i.e., mammals, birds, reptiles, and fishes) but also in invertebrates (i.e., *Anopheles*).
